# Primary healthcare as a strategy for eliminating hepatitis C: the METRIC toolkit

**DOI:** 10.1017/S1463423624000355

**Published:** 2024-11-07

**Authors:** Ricardo Baptista-Leite, Henrique Lopes, Diogo Franco, Timo Clemens, Helmut Brand

**Affiliations:** 1 NOVA Center for Global Health – NOVA Information Management School (NOVA IMS), Universidade Nova de Lisboa, Lisbon, Portugal; 2 Department of International Health, Care and Public Health Research Institute – CAPHRI, Faculty of Health, Medicine and Life Sciences, Maastricht University, Maastricht, The Netherlands

**Keywords:** hepatitis C, hepatitis C elimination, general practitioners, primary healthcare

## Abstract

**Aim:**

This paper presents the development of the METRIC toolkit, aimed at enhancing primary healthcare interventions in the context of hepatitis C control, thus contributing to the World Health Organization’s global strategy to achieve the elimination of the disease by 2030.

**Background:**

At the global level, most people living with hepatitis C are unaware of their condition. As such, the eradication of hepatitis C necessitates comprehensive strategies within primary healthcare settings, as it provides an opportunity to reach the general population, facilitates the identification of potential patients who may be unfamiliar with hepatitis C, and guides them toward adequate care. Herein, we propose the METRIC toolkit as a means to optimize the efficiency and efficacy of healthcare services dedicated to hepatitis C control.

**Methods:**

The development of the METRIC toolkit was guided by a thorough review of pertinent literature, focusing on primary healthcare interventions in hepatitis C control. Key components were identified, encompassing systematic problem identification, solution formulation, outcome evaluation, and feedback integration.

**Findings:**

The METRIC toolkit represents a valuable resource for strengthening primary healthcare interventions in hepatitis C control. By fostering a culture of continuous improvement and data-driven decision-making, this framework holds promise in advancing the global agenda towards hepatitis C elimination. However, its successful application requires careful consideration of contextual factors and ongoing adaptation to local needs and circumstances.

## Background

The Alma Ata Declaration (WHO, 1978) states the importance of primary healthcare as the first line of contact with the population and in the effective combat of diseases. By having a significant impact on supporting the population, primary healthcare is also a cost-effective approach to guaranteeing health quality (WHO, 2018).

Since the Alma Ata Declaration, the structuring of healthcare worldwide transformed primary healthcare into an important tool to improve healthcare levels and reduce morbidity profiles (OECD, 2020; WHO, 2022). In this process, more complex interventions are reserved for secondary and tertiary care due to the need for more expensive complementary diagnostic and treatment resources for specific clinical situations that cannot be addressed in primary healthcare. Amidst the commemorations of the forty years of the Alma Ata Declaration, the World Health Organization (WHO) recently reinforced the political commitment to primary healthcare to guarantee the best possible attainable ‘Health for All’ worldwide (WHO, 2019).

### Hepatitis C

The hepatitis C virus (HCV) often leads to infections that trigger life-threatening comorbidities and severe hepatic complications (Veracruz *et al*., 2022). The mortality related to the disease was estimated to be around 290,000 deaths in 2019 (WHO, 2022). The risk of hepatocellular carcinoma in patients with cirrhosis is between 10% and 40% per decade, setting it as one of the deadliest cancers in terms of individual prognosis (Sharma *et al*., 2018).

Globally, chronic infection with HCV has a high prevalence and is among the seventh leading cause of death (Stanaway *et al*., 2016), being identified as a significant Public Health issue. Around the world, it’s estimated that 58 million individuals live with chronic HCV infection, and approximately 1.5 million new cases are reported annually (WHO, 2023). Although the disease seems to be endemic, there is a considerable variation in the geographical prevalence of HCV, with estimates suggesting that around 75% of the HCV burden is concentrated in 20 countries (Gamal & El Kassas, 2021).

The HCV infection exhibits a heterogeneous distribution globally, reflecting variations in prevalence both across and within countries (Guntipalli *et al*., 2021). The Eastern Mediterranean region exhibits the highest HCV prevalence at 2.3%, surpassing the WHO European region, which has a prevalence of 1.5%. This is due to the case of Egypt, which poses a distinctive challenge regarding the prevalence of HCV. Recent data showed an overall prevalence rate of 4.6% for HCV antibodies and 3.5% for active HCV infection among approximately 50 million Egyptians from 2018 to 2019. This has led to a significant burden of chronic HCV infection in the country, resulting in a rise in cases of liver cirrhosis, portal hypertension, and hepatocellular carcinoma in recent years (Waked *et al*., 2020). Conversely, the South-East Asia region reports the lowest prevalence, marked at 0.5% (Roudot-Thoraval, 2021). Subsequent research in 2022 (Goel *et al*., 2022) indicates that the seroprevalence and disease burden of HCV in the SEAR are comparatively lower than in other WHO regions, probably due to the lack of diagnoses in many of the most fragile populations.

The primary HCV transmission source is blood-borne, for instance, through sexual transmission and among populations at higher risk, such as people who inject drugs (PWID), prisoners, and others (Parsons, 2022). Worldwide, hospital-acquired infections are linked to practices that lack adequate infection control, including blood transfusion and long-term haemodialysis (Jadoul *et al*., 2019), the use of the same syringe for multiple individuals, and the reuse of glass syringes (Kim, 2016), among other healthcare materials and practices. Furthermore, the ongoing importance of nosocomial infection in high-income countries should be noted, emphasizing the need for continued vigilance and improvement in infection control measures across all healthcare settings (Jadoul *et al*., 2019). Additionally, HCV in the community can also be traced back to historical exposure through contaminated blood, highlighting the critical need for stringent infection control and safe blood transfusion practices to prevent such infections.

Although less common, mother-to-infant transmission also occurs, with an associated risk between 2% and 11% (Bernstein *et al*., 2018). Other less-studied groups also appear to be populations at higher risk, particularly war veterans and individuals who acquired tattoos with no proper care measures for inking material (van Dooren *et al*., 2014).

The silent nature of HCV infection often leads to it going unnoticed, as it can remain asymptomatic in patients for several decades (Dopico *et al*., 2022). This asymptomatic characteristic significantly hinders the disease’s control, as individuals living with HCV infection unknowingly compromise their health over time and become potential vectors for transmission (Ray & Ray, 2019). Furthermore, the risk behaviours associated with HCV transmission, potentially occurring decades prior, may be forgotten or underappreciated, diminishing the perceived risk and urgency for testing and treatment in the present.

This issue is compounded by the barriers to effective prevention and rapid treatment, including limited access to healthcare, social stigma, and a lack of awareness both among the general public and healthcare professionals. As a result, many individuals do not receive timely diagnosis or care, leading to preventable complications and the further spread of the virus. To address this, primary healthcare can play a crucial role by conducting retrospective risk analyses in populations currently not perceived as being at risk, thereby identifying undiagnosed cases and facilitating early intervention (Kasting *et al*., 2020). Literature supports the importance of overcoming these barriers through enhanced screening (Schillie *et al*., 2020), awareness campaigns (IOM Egypt, 2019; Etoori *et al*., 2023), and the integration of HCV testing into routine healthcare practices to reduce the disease’s morbidity and mortality rates (Coyle *et al*., 2016). By focusing on these strategies, healthcare systems can significantly reduce the impact of HCV as a public health issue, demonstrating that much of the morbidity and mortality associated with HCV is amenable with proper attention to prevention and early treatment.

The significance of HCV mortality and comorbidity led the World Health Organization to define a global strategy in 2016 for the elimination of the disease by 2030, by reducing new infections by 90% and deaths by 65%. The target comprised a set of goals aiming to reduce the number of new cases by 90%, reaching 90% of diagnosed cases, having at least 80% of patients undergoing treatment, and reducing mortality by 65% by 2030, among others (WHO, 2016). However, only 11 countries are on track to achieve HCV elimination by 2030 (Polaris Observatory, 2022).

### Why is primary care important?

Globally, the decentralization of HCV treatment to primary healthcare remains limited, risking the overburdening of specialty services in high-prevalence areas, which could hinder optimal treatment outcomes (Oru *et al*., 2021). Unlike advanced liver disease cases, HCV treatment generally doesn’t require tertiary care, and recent advancements have pushed cure rates above 95% (Cornberg & Manns, 2022). Without tailored national HCV programs, reflecting the epidemiological landscape, prevalence and mortality may rise, particularly in lower-income countries. This highlights the need for integrating HCV care into primary health settings to improve accessibility and efficiency.

Primary care offers extensive benefits in healthcare delivery, notably its broad population coverage and lower barriers to access. This accessibility fosters a high level of trust between patients and healthcare providers, who possess a deep understanding of the local community’s health needs. Additionally, primary care excels in reaching out to marginalized and stigmatized populations, providing a crucial platform for the early detection, reporting, and follow-up of acute infections (Wade *et al*., 2020).

For patients, the benefits of engaging with primary care include the comfort and confidence that come with working with a trusted team of known healthcare professionals. This familiarity significantly reduces the treatment burden on patients by streamlining their care experience. Moreover, primary care acts as a gateway to community services, offering practical, social, and material support. This comprehensive approach not only addresses medical needs but also enhances the overall well-being of patients by connecting them with essential community resources (Stewart *et al*., 2023).

Additionally, despite the ecological footprint of healthcare being notably high (and the methods to accurately measure this being currently limited), primary care presents a greener alternative, as its ecological footprint is significantly smaller compared to secondary or tertiary settings. This reduction is largely due to decreased patient travel requirements to centralized healthcare facilities, underscoring the environmental benefits of localizing care within primary care settings (Nicolet *et al*., 2022).

The valorization of this first level of contact with the health system becomes a pivotal strategy in combating HCV (Cabezas *et al*., 2022; Crespo *et al*., 2023, 2020b, 2020a), particularly due to its capacity to address the multifaceted challenges of chronic diseases, including asymptomatic conditions. The integration of longitudinal patient records and patient registers within primary care settings underpins the ability to identify and manage patients over time, thereby playing a crucial role in tracking health changes and facilitating appropriate interventions early on. These foundational tools offer a comprehensive overview of a patient’s health history, crucial for identifying diseases in their silent stages.

Addressing the barriers to care access for individuals from endemic countries or those with unscrutinized exposure to HCV through blood transfusions is another domain where primary healthcare proves indispensable (Bansal *et al*., 2023). Obstacles such as racism, administrative hurdles, cost, and practical issues often impede the detection and care of HCV among at-risk populations. The implementation of inclusive, non-stigmatising strategies within primary care settings is vital, ensuring that all individuals, irrespective of their background or circumstances, have equitable access to healthcare services (Bhatnagar & Canzater, 2022).

In some regions, directly integrating HCV care within primary healthcare settings may not be feasible due to resource limitations or regulatory frameworks. In these instances, alternative solutions such as the ECHO project, telemedicine tools, and electronic alert systems play a crucial role. These technologies enable primary care providers to consult with specialists and manage care remotely, effectively bridging the gap in healthcare delivery. Such systems ensure that individuals who require specialized care can receive timely interventions, thereby improving linkage to care and supporting continuous management of hepatitis C across different healthcare settings (Arora *et al*., 2017; Mendizabal *et al*., 2019; Crespo *et al*., 2023).

Understanding the opportunities for intervention across the cascade of care for HCV is crucial, particularly in highlighting the role of primary healthcare services in HCV elimination. Primary care can serve as a pivotal entry point for HCV screening, diagnosis, and linkage-to-care, effectively addressing the virus at multiple stages. By leveraging primary healthcare’s accessibility, HCV management can become more proactive and patient-centred, ensuring early detection, timely treatment initiation, and follow-up care. This approach not only streamlines the pathway to HCV elimination but also significantly reduces the disease burden on tertiary healthcare facilities.

The concept of cascade of care had no single universally accepted definition in the literature. For this article, it was adapted from the Global Hepatitis Report (WHO, 2017) and the consensus HCV cascade of care (Safreed-Harmon *et al*., 2019) to align with the context of primary healthcare. Therefore, this reading comprises the cascade of care dimensions of awareness and prevention, testing, linkage-to-care, and treatment.

### Awareness and prevention

Populations at higher risk are often assisted in primary healthcare for a variety of health problems due to the conjoint work with specialized services (PWID assistance with treatment services, primary healthcare chains, and primary healthcare in places with a high density of sex workers) for both primary and secondary prevention.

Primary healthcare plays a critical role in the awareness of risks related to HCV which populations might incur due to lifestyle, as well as in the prevention of contracting the disease through risk behaviours. By maintaining continuous engagement with a significant portion of the population, primary healthcare stands as one of the most suitable healthcare systems for identifying and documenting individuals with risk behaviours and pathologies, often linked to hepatitis C (Rogal *et al*., 2017; Lopes *et al*., 2022), especially those who are unaware of their HCV infection. The primary goal is to offer counselling, facilitate testing, establish linkage-to-care, and provide treatment to mitigate transmission pathways (WHO, 2022).

Primary healthcare and specialized services should concurrently implement secondary prevention strategies. This involves providing tailored health literacy education and counselling for individuals with HCV. The focus is on empowering patients to manage their health effectively, emphasizing the reduction of transmission risks through informed behavioural choices and supportive policy reforms. This is particularly vital for groups at higher risk of reinfection, such as PWID and incarcerated individuals.

Moreover, it is essential to enhance collaboration between sexual and reproductive health services and primary care. This integration aims to extend comprehensive care and prevention efforts to include those who may not self-identify within specific at-risk groups o who encounter barriers to accessing healthcare services, ensuring no one is left behind (Jin *et al*., 2021).

Additionally, secondary prevention efforts should prioritize education on preventive practices and policy-driven changes to support individuals in managing their health post-treatment. Effective strategies focus on minimizing behaviours associated with increased risk and reinforcing policies that support sustained health outcomes in populations known to experience higher rates of infection, including PWID and incarcerated individuals (WHO, 2018; Han *et al*., 2019).

### Testing

Identifying people at risk of acquiring HCV infection is the ﬁrst step towards improving health outcomes and providing counselling opportunities on how to reduce infection risks. The testing process was considered to be fundamentally comprised of two key components: screening and diagnosis. This strategy expects that the associated morbidity and mortality may be reduced by identifying and diagnosing the disease before the onset of symptoms. Considering that the majority of individuals living with hepatitis C are yet to be identified, large-scale interventions are necessary to control the disease at a global level. Interventions recommended by international health entities of reference such as the WHO, the Centre for Disease Prevention and Control (CDC), and the European Centre for Disease Prevention and Control (ECDC) are only affordable if implemented through proximity services, such as primary healthcare. To address this issue, the ECDC (ECDC, 2022) and the WHO (WHO, 2022) recommend testing for hepatitis C in key populations due to high-risk behaviours that may lead to contracting infectious diseases, such as PWID, MSM, prisoners, sex workers, transgender individuals, among others. Although having common recommendations with the ECDC and the WHO, the CDC (Schillie *et al*., 2020) also points to the need to provide testing considering different dimensions under specific conditions: 1. universal HCV screening; 2. one-time HCV testing for individuals with backgrounds or lifestyles that may incur the risk of contracting hepatitis C; 3. routine HCV testing for individuals that incur risk behaviours; and 4. HCV testing for anyone upon request.

The possibility of associating screening of more than one pathology in a single act has the potential of achieving multiple benefits in situations such as the inclusion of diagnostic tests in a battery of tests associated with specific ages, specific conditions, such as pregnancy, and the vigilance of other diseases, among others. Also, conducting HCV screenings in primary healthcare vastly reduces the cost of medical acts, and lowers both the patient’s effort and the demand burden on Health Services (Munang *et al*., 2019). This approach allows for: a) a more global assessment instead of focusing on a single pathology; b) the dilution of the emotional weight of each isolated diagnosis, placing hepatitis C at the same level as other diseases and minimizing stigma; c) avoids situations that may lead to patient dropout or non-compliance regarding diagnostic tests, ranging from fear of results, lack of motivation by having to travel to the test site, and fear of needles, among others (Shehata *et al*., 2018).

### Linkage-to-care

The ultimate goal in the cascade of care is to ensure a maximum number of individuals progress through each stage of the process successfully. Linkage-to-care includes key dimensions that are crucial for achieving the 2030 elimination target, as highlighted in the EMCDDA (2019) report. Nevertheless, existing literature reveals that linkage-to-care remains a critical step, with approximately 30% of individuals who test positive for HCV antibodies not proceeding to confirmatory tests or specialty services, as reported by Strebe *et al*. (2022).

Extensive research into linkage-to-care challenges, particularly during the pre-direct-acting antiviral era, is documented in HCV guidelines. These challenges encompass various factors, including the presence of comorbid conditions, conflicting priorities, high rates of loss to follow-up, prolonged treatment durations with associated adverse effects, limited treatment accessibility, and a shortage of practitioner expertise (EMCDDA, 2021).

Potential strategies were also identified to surpass the imposed challenges and increase linkage-to-care, such as the promotion of initiatives of education and counselling, allowing co-localization of the HCV cascade of care in medical (e.g., primary healthcare services) or social programmes (e.g., Needle and Syringe Programmes, Opioid Substitution Treatment) (AASLD-IDSA, 2017; Schwarz *et al*., 2022). Education and counselling for individuals at higher risk of infection and agents that may participate in the cascade of care, such as health professionals or peers (workers and non-workers), can improve knowledge regarding the therapeutical process of HCV (Batchelder *et al*., 2017; Marco *et al*., 2022).

In recent guidelines published by the WHO, it is recommended that along with testing for HCV, services of linkage to prevention, healthcare, and treatment must also be offered, especially for most affected populations (WHO, 2022). As a major part of the population is regularly assisted in primary healthcare, it becomes easier not to lose people in the treatment process. Furthermore, this allows for an integrated health response to be offered, in that the primary healthcare practitioners can address the patient’s set of conditions, which often goes beyond the intervention capacity of highly differentiated services, which are specialized in an area (Whiteley *et al*., 2022).

### Treatment

The introduction of direct-acting antivirals represents a groundbreaking shift in the HCV treatment landscape, fundamentally altering the treatment paradigm. This innovation has made it possible to administer this therapy not only in secondary or tertiary healthcare settings but also within the scope of primary healthcare services.

While there’s a proposal to shift the approach to hepatitis C towards primary healthcare, it doesn’t negate the importance of maintaining collaboration between primary healthcare, secondary, and even tertiary services when circumstances warrant it. This is especially relevant when patients have concurrent health issues alongside their HCV infection. In such situations, the objective is to foster a matrix-style approach, where primary healthcare has its dedicated role and intervention area (Whiteley *et al*., 2022).

Considering that hepatocellular carcinoma is among the leading global causes of mortality, it is imperative to establish a proactive strategy for its risk reduction. This strategy should involve regular surveillance, particularly for individuals at elevated risk, such as those with cirrhosis or advanced hepatic fibrosis. It is important to note that this risk remains even for individuals who have achieved sustained virologic response (SVR) through direct-acting antiviral therapy (Moghe and Shaikh, 2018).

Recent developments in the healthcare approach to HCV are centred around streamlining the entire cascade of care process, with a primary focus on reducing the risk of patients being lost during transitions (van Dijk *et al*., 2020; Krekulova *et al*., 2022). Recognizing the pivotal role that primary healthcare plays in managing HCV, given its wide reach and engagement with a substantial population, it becomes essential to bolster the availability and delivery of direct-acting antiviral therapies within these primary healthcare services. This strategic move is critical in the pursuit of the WHO’s HCV elimination objectives (EMCDDA, 2021; WHO, 2022).

In light of the aforementioned factors, the objective of this article is to explore potential avenues for the involvement of primary healthcare services in the comprehensive management of HCV across various stages of the disease cascade of care. These stages encompass awareness and prevention, testing, linkage-to-care, and treatment. To facilitate this endeavour, we introduce an action toolkit known as METRIC^1^, designed to facilitate continuous quality enhancement in HCV care within the primary healthcare setting.

The METRIC toolkit is designed to support the WHO’s goal of eliminating hepatitis C by 2030. One of its major strengths lies in enabling the implementation of complex intervention that support not just the typical patient but also marginalized groups such as drug users, people from endemic regions, and those infected via healthcare-related transmission. This includes strategies to prevent mother-to-baby transmission by ensuring that women are identified and treated early.

The METRIC toolkit is designed to assist in the identification of challenges and potential solutions while also providing the means to analyse outcomes. This analytical approach enables necessary adjustments within the primary healthcare ecosystem. Ultimately, METRIC serves as a scientifically oriented action toolkit aimed at enhancing the overall effectiveness of HCV management in primary healthcare settings.

## Materials and methods

**Figure 1.**
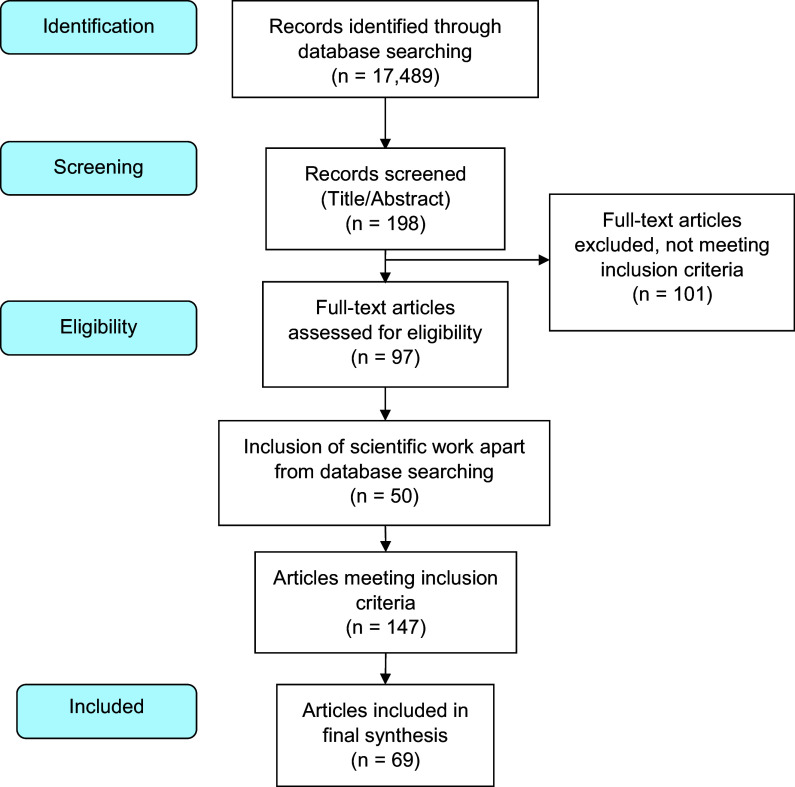
Flow diagram of the study selection aggregated over the topic areas reviewed according to inclusion and exclusion criteria.

The present manuscript presents a scoping review and thematic content analysis focusing on primary healthcare services in the context of the HCV cascade of care, with an exclusion of chronic care and social rehabilitation due to the specific emphasis on disease-centric care rather than post-cure management. Data were systematically gathered from prominent academic databases including ‘Scopus’, ‘Google Scholar’, and ‘PubMed’, and structured according to the Preferred Reporting Items for Systematic Reviews and Meta-Analyses (PRISMA) guidelines (Figure 1).

An initial comprehensive search utilized key terms such as ‘Hepatitis C’, ‘Primary healthcare’, and ‘Primary Care’. Followed by refined searches targeting aspects of ‘Hepatitis C management’ and ‘primary care models for Hepatitis C’, yielding a total of 17,489 hits. After screening abstracts and titles, eliminating duplicates and inaccessible articles, 198 relevant publications were identified. Subsequent application of inclusion and exclusion criteria (Table 1) resulted in the selection of 97 articles, as delineated in Figure 1. Additionally, literature emanating from a Think Tank involved in creating the ‘National Strategic Consensus for Hepatitis C’, in 2014 (Baptista-Leite *et al*., 2014) was incorporated, which involved consultation with twenty-seven experts over ten months. Correspondingly, insights from the National Advisory Board of the Hepatitis C project named ‘Let’s End Hepatitis C’ were also considered. The amalgamation of data from these sources culminated in the selection of 147 pertinent articles, from which 69 were utilized in the current study.

**Table 1.** Inclusion and exclusion criteria for scientific works included in the article

**Inclusion criteria:**
Present in the results from the searches focused in ‘Hepatitis C’, ‘Primary Health Care’, ‘Primary Care’ and ‘Management of Hepatitis C’.
Article focus in PHC settings applied to hepatitis C care.
Publication and information collection performed after 2013.
**Exclusion criteria:**
Publications or data previous to 2013.

Subsequent data analysis, guided by the scoping review, entailed qualitative examination, integrating expert perspectives aligned with recommendations from the ‘Let’s End Hepatitis C’ project concerning public health strategies tailored to hepatitis C management within primary healthcare settings. This analysis substantiated the development of the METRIC framework, facilitating the identification of cations conducive to continual enhancement of quality in primary healthcare provision for HCV. The METRIC toolkit is delineated through a decision-making flowchart, facilitating a broad comprehension of organizational processes and aiding in problem identification and resolution in line with best practices and desired outcomes. By fostering a culture of continuous quality improvement, METRIC endeavours to mitigate the prevalence of HCV by optimizing healthcare processes within specific ecosystems, thereby offering evidence-based guidance and bolstering external audit mechanisms.

## Development of the METRIC toolkit for strengthening primary healthcare interventions in HCV control

The METRIC toolkit is proposed to bolster primary healthcare interventions in the HCV cascade of care, aiming to advance the goal of HCV elimination by enhancing the efficiency and efficacy of healthcare services. Informed by a literature review, five main components were identified for the toolkit construction: Problems, Solutions, Outputs, Outcomes, and Feedback Process, based on the Donabedian Plan-Do-Check-Act (PDCA) method. The core concept emphasizes continual knowledge generation and response refinement through iterative cycles, allowing for continuous improvement via registry, analysis, and accountability. However, its usability is contingent upon specific country contexts, including social, economic, and legal factors, necessitating selective implementation of feasible points.

**Figure 2.**
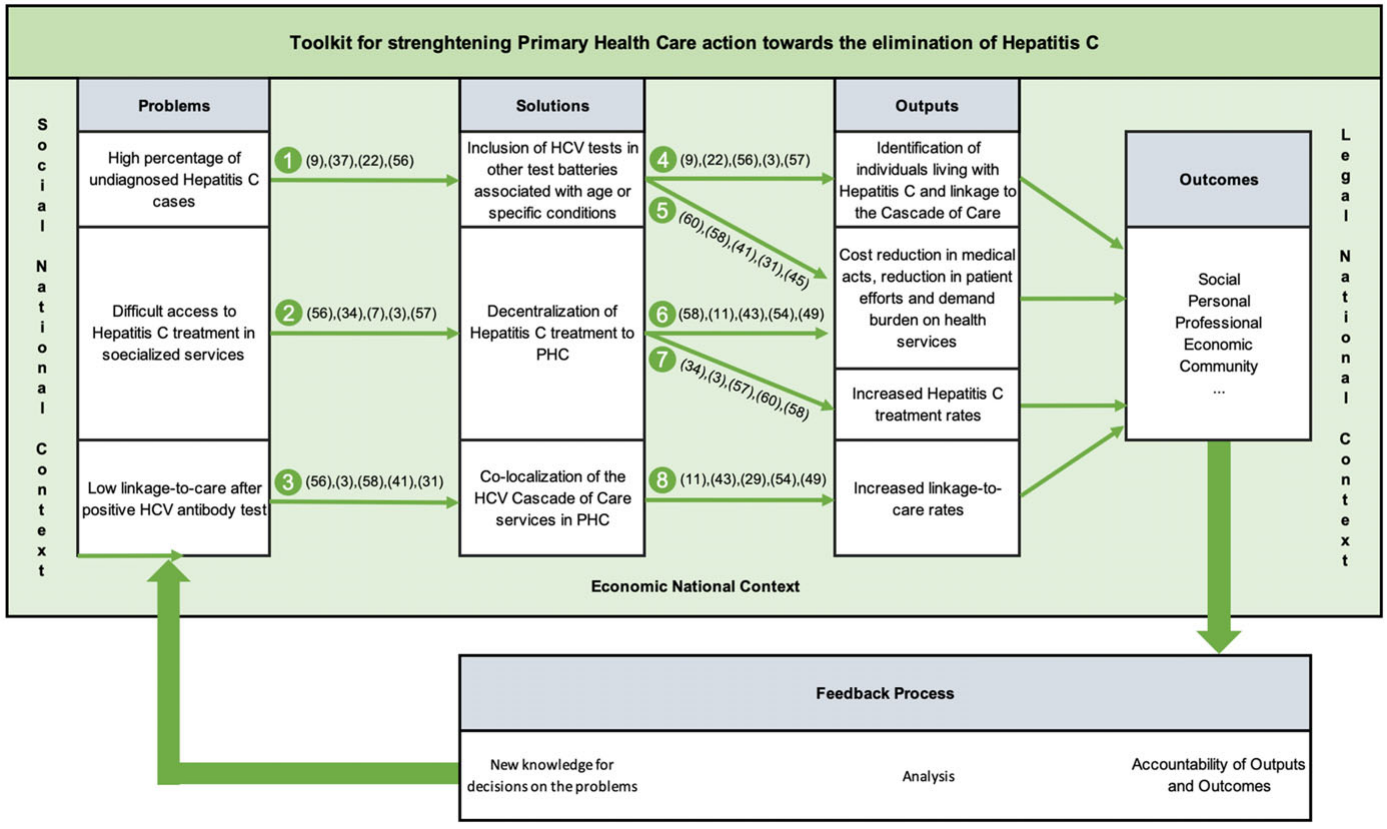
Proposal of a toolkit to strengthen Primary Health Care action towards the elimination of hepatitis C – METRIC.

Intervention toolkits for primary healthcare should prioritize intelligence through data collection, processing, and storage, crucial for generating new solutions and optimizing procedures. This approach involves correcting deviations through gap analysis or creating new paths pertinent to the analysed ecosystem. Implementation requires tailored written procedures for each of the eight process procedures depicted in Figure 2, including adaptation to the organization’s ecosystem and legal framework, delineation of responsibilities, quantification of activities, and inclusion of an audit system for deviation identification and correction.

An essential component of the METRIC toolkit’s implementation involves collaboration across various sectors of the healthcare system. The toolkit provides a structured framework wherein primary care, public health entities, specialist teams, and community services can effectively integrate their efforts. This integrative approach is particularly advantageous when supported by ring-fenced funding, allowing for a coordinated action plan that aligns with the WHO’s directives for hepatitis C elimination.

That said, the METRIC toolkit can be applied across various levels of HCV control, ranging from individual Health Centers, Health Insurance Companies, or other small-scale facilities tasked with providing follow-up care aimed at mitigating the disease burden associated with HCV in their respective populations, to larger regional or national structures seeking specific outcomes. These outcomes may include but are not limited to: reducing costs associated with HCV by shifting care from secondary and tertiary levels to primary care settings; enhancing the productivity of primary healthcare teams by broadening their scope of patient engagement; and identifying ‘hidden patients’ who are unaware of their HCV infection status and are not typically associated with high-risk groups, among other objectives.

The implementation of the METRIC toolkit is straightforward and can be approached either holistically for optimal overall outcomes or in segmented portions if specific structural issues have been identified. In the latter scenario, simply following the directional cues provided in Figure 2 facilitates the identification of subsequent steps.

It is highly recommended that the METRIC toolkit be integrated with Health Quality Policies (HQP), as this synergy fosters mutual enhancement: established procedures within HQP greatly facilitate the development of tailored procedures for each operating unit, ensuring alignment with the unit’s existing workflow and organizational context. Conversely, alignment with HQP aids in comprehending the rationale behind the Plan-Do-Check-Act (PDCA) methodology, a fundamental component of operations in all health units that prioritize quality in health as a core strategic element for achieving desired outputs and outcomes. A robust integration between the METRIC toolkit and the chosen PQS (Quality System) within the health structure enhances synergistic effects, yielding improved outcomes in METRIC and health procedure outputs.

That said, to effectively implement the METRIC toolkit, it is recommended that stakeholders from all levels of healthcare delivery come together to form a unified front. This includes securing dedicated funding to ensure that all elements of the toolkit can be deployed, thus enabling primary care facilities to lead the charge in prevention, early detection, and management of hepatitis C. Such a strategy not only addresses the direct needs of the affected populations but also supports the broader public health goal of disease elimination.

## Discussion and conclusions

In recent decades, there has been a growing push to engage citizen more actively in healthcare processes, a concept evolving into a more inclusive practice that involves people with lived experiences in the codesign of primary care systems. This approach extends beyond the traditional ‘Citizenship for Health’ (Lopes & McKay, 2020) by ensuring that all residents, regardless of citizenship status, are included in the health discourse. The World Health Assembly recently endorsed a resolution on social participation in health recognizing the critical role of diverse community involvement in shaping health services that are responsive to the needs of all individuals and families (WHO, 2024). To empower communities effectively, it is imperative to foster enhanced awareness of health issues and encourage broad-based participation. This initiative involves not only acquiring knowledge and translating it into tangible policies but also enhancing the management of the healthcare system’s responsiveness through the active engagement of those directly affected by health policies. By boosting health literacy and promoting inclusive participation, we can significantly enhance preventive care and raise awareness of the risks individuals face and may pose to others. This inclusive approach fosters participation in crucial decision-making processes, leading to better adherence to therapeutic regimens and improved communication between patients, caregivers, and their healthcare providers (Lopes, 2018). Ultimately, this collaborative effort, free from conflicts of interest related to pharmaceutical funding, facilitates a more informed and effective healthcare delivery system that truly reflects the needs of its diverse population.

Especially in the realm of infectious diseases like HCV, heightened awareness and activism for health causes are of paramount importance. They yield significant individual and collective contributions, fostering risk awareness, facilitating diagnosis, and managing therapy among populations that are often particularly vulnerable. Such efforts also play a crucial role in establishing the broader frameworks needed to generate positive clinical outcomes.

Addressing a disease like HCV comprehensively may necessitate not only medical protocols but also social and legal support tailored to the patient’s needs. In this regard, primary healthcare stands out as particularly suited to the patient’s environment compared to secondary and tertiary health services. In the context of HCV, this proximity is invaluable across all stages of the cascade of care. It facilitates preventive measures among at-risk groups and enables the identification of new cases through opportunistic screening, especially within marginalized social segments that may not regularly seek healthcare, such as migrants, the unemployed, individuals with dependencies, and communities distant from major urban centres where specialized care is less accessible.

The shift of HCV management from in-hospital to outpatient settings, facilitated by the advent of direct-acting antiviral treatments and pangenotypic medications, has simplified therapeutic regimens and enabled greater involvement in primary healthcare. Despite the diverse nature of primary healthcare systems worldwide, they share the commonality of maintaining contact with a significant portion of the population, making them pivotal in streamlining the HCV cascade of care.

To achieve the goal of eliminating HCV by 2030, structural reforms integrated with national strategies are imperative. This necessitates proactive measures, including the integration of clinical guidelines into practice, supported by adequate human and financial resources, as well as political commitment. A key aspect of combatting HCV lies in identifying individuals living with the disease, as a substantial proportion remain undiagnosed even in developed countries. The ongoing engagement between primary healthcare services and the population facilitates the identification of high-risk individuals and ensures their timely linkage-to-care through streamlined interventions.

The evolution of primary healthcare in managing HCV, in alignment with recommendations from entities such as the European Association for the Study of the Liver, the World Health Organization, and the American Association for the Study of Liver Diseases, will play a pivotal role in identifying millions of HCV cases, saving lives, and preventing millions of new infections.

The proposed METRIC toolkit seeks to bolster primary healthcare’s role across all stages of the HCV cascade of care, thereby contributing to the goal of disease elimination by 2030. By transitioning HCV management to primary healthcare, there is potential to reduce disease prevalence and healthcare costs. This approach enables primary healthcare to implement comprehensive awareness campaigns, educate individuals on lower-risk behaviours, and mitigate primary healthcare incidence and reinfection rates, particularly during reintegration into society. Additionally, a primary healthcare approach allows for more thorough patient history assessments, enhanced follow-up programmes, and improved detection of cases that may fall outside the traditional healthcare system’s purview, especially within high-risk populations.

## Footnotes

1 METRIC: Pri**M**ary h**E**al**T**hca**R**e Hepat**I**tis **C**
